# “Iluminando o Coração” com Eco 3D: Transiluminação de Deiscência de Prótese Valvular Mitral

**DOI:** 10.36660/abc.20210655

**Published:** 2022-07-29

**Authors:** Mariana Ribeiro Silva, Ana Isabel Azevedo, Francisco Sampaio, José Ribeiro, Ricardo Fontes-Carvalho

**Affiliations:** 1 Centro Hospitalar de Vila Nova de Gaia/Espinho Porto Portugal Centro Hospitalar de Vila Nova de Gaia/Espinho, Porto – Portugal; 2 University of Oporto Oporto Portugal University of Oporto, Oporto – Portugal

**Keywords:** Ecocardiografia, Tridimensional/métodos, Doenças Cardiovasculares/diagnóstico por imagem, Doenças cardiovasculares/fisiopatologia, Interpretação de Imagem Assistida por Computador, Variações Dependentes do Observador, Transluminação, Insuficiência da Valva Mitral/diagnóstico

O ecocardiograma tridimensional (3D) com transiluminação (TI) é uma nova ferramenta de renderização 3D que melhora características específicas de uma imagem que não são exibidas de maneira ideal por imagens em 3D convencionais. A renderização 3D convencional não tem a consistência, em relação a detalhes da imagem e percepção de profundidade, geralmente apresentando imagens inadequadas. Imagens 3D com transiluminação aproveitam a integração de uma fonte de luz virtual móvel ao conjunto de dados. A fonte de luz pode ser movimentada para frente e para trás e de lado a lado, sendo posicionada em locais específicos para destacar a região de interesse, aumentar a precisão, melhorar a percepção de profundidade, criar sombras e permitir uma distinção mais precisa entre estruturas. Além disso, essa nova ferramenta de renderização 3D melhora a visualização e o delineamento de orifícios e bordas, cavidades, massas e anormalidades estruturais^1,2^ e é essencial para captar imagens detalhadas durante procedimentos.^3^

A transiluminação pode ser particularmente valiosa em cenários desafiadores, especialmente na avaliação de próteses e dispositivos cardíacos que produzem sombreamento acústico, levando a uma maior precisão diagnóstica.^4,5^

No painel, apresentamos dois casos em que o ecocardiograma 3D com TI apresentou maior valor diagnóstico na avaliação de deiscência de prótese valvular.

## Caso 1

**Figura 1:**
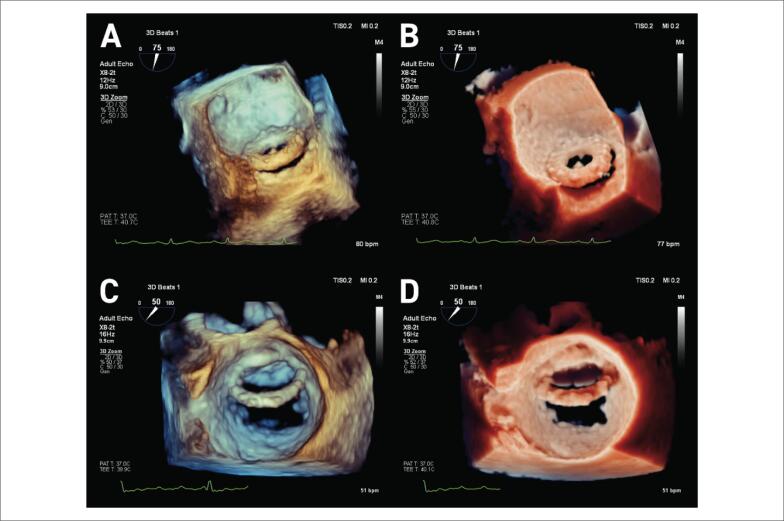
Painéis A e B. Deiscência da prótese mitral 3M Starr-Edwards vista do átrio esquerdo (apêndice atrial esquerdo na posição de 9 horas no relógio). Painel A: imagem 3D convencional. Painel B: Renderização com TI, a luz é posicionada abaixo do apêndice atrial esquerdo. O efeito de sombreamento melhora a percepção de profundidade e oferece uma definição mais precisa do grau de deiscência protética. Painéis C e D: Deiscência do anel mitral vista do átrio esquerdo (válvula aórtica entre a posição de 11 horas e 12 horas no relógio). Painel C: imagem 3D convencional. Painel D: Renderização com TI; a luz é posicionada lateralmente próximo ao apêndice atrial esquerdo, melhorando os pontos de separação do anel e retratando a integridade do folheto mitral posterior. 3D: tridimensional; TI: transiluminação.

Um paciente do sexo masculino de 55 anos de idade passou por um implante de prótese bola-gaiola (3M Starr-Edwards) aos 29 anos de idade. Ele foi admitido com insuficiência cardíaca aguda, classe funcional III da New York Heart Association (NYHA). O ecocardiograma transtorácico (ETT) demonstrou vazamento periprotético mitral de moderado a grave; o ecocardiograma transesofágico (ETE) em 3D demonstrou deiscência da prótese, com vazamento periprotético grave (Figura 1A). A TI melhorou a percepção de profundidade e a definição precisa do grau de deiscência protética, demonstrando uma desinserção da prótese mitral envolvendo mais de 50% da circunferência mitral (Figura 1B; Vídeo 1).

**Vídeo 1:**
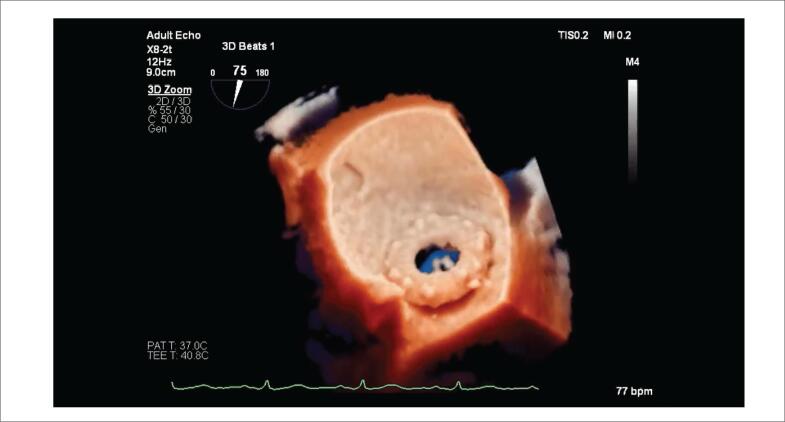
URL: http://abccardiol.org/supplementary-material/2022/11902/2021-0655-video-1.mp4

## Caso 2

Um paciente do sexo masculino de 73 anos de idade passou por anuloplastia mitral com um anel completo de 34 mm. Quatro anos mais tarde, ele apresentou dispneia. O ETT revelou regurgitação mitral (RM) moderada e uma estrutura hiperecogênica no átrio esquerdo; o ETE-3D confirmou a presença de RM moderada e mostrou um anel parcialmente separado (Figura 1C). Nesse caso, a TI permitiu uma melhor visualização dos pontos de separação da prótese em anel, demonstrando a integridade do folheto mitral posterior e forneceu informações adicionais sobre o mecanismo de RM, que se devia a um anel mitral nativo dilatado que levou a uma coaptação incompleta do folheto (Figura 1D, Vídeo 2).

**Vídeo 2:**
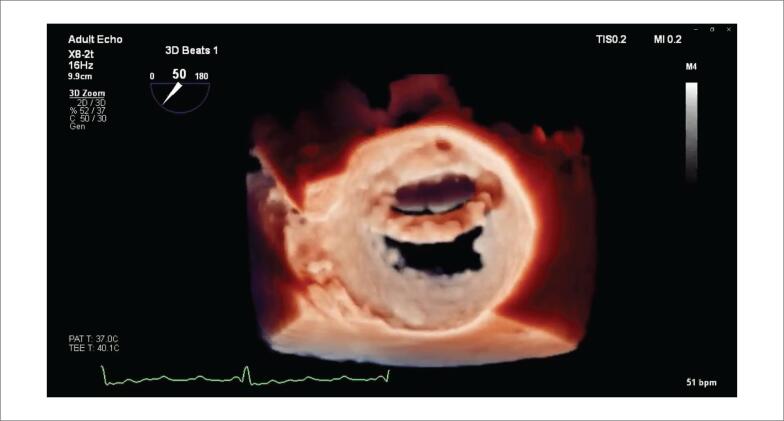
URL: http://abccardiol.org/supplementary-material/2022/11902/2021-0655-video-2.mp4

Apesar de a TI 3D ser altamente viável em uma variedade de doenças cardíacas, incluindo a doença cardíaca estrutural, ela ainda não é uma técnica amplamente disponível, exige treinamento adequado e aprofundamento nos estudos com foco em desfechos clinicamente relevantes e na eficácia em validar a implementação da renderização por TI na prática clínica de rotina. Além disso, as evidências atuais ainda estão limitadas em relação aos benefícios agregados da TI em comparação com outras técnicas.

Ainda assim, essa nova técnica não exige uma curva de aprendizado muito íngreme e é um processo relativamente intuitivo para mover a fonte de luz virtual para enfatizar a estrutura de interesse.^4^ A transiluminação surge como uma alternativa à imagem 3D convencional, especialmente nas condições em que se imagine que a renderização convencional vai produzir imagens inadequadas, especialmente na avaliação da doença de prótese valvular.^5^

Esses dois casos destacam a importância da renderização por TI na avaliação de doença cardíaca estrutural complexa.
