# Supplementary material for: “Iluminando o Coração” com Eco 3D: Transiluminação de Deiscência de Prótese Valvular Mitral
Source: Arq Bras Cardiol. 2022 Jul 29;119(2):358–60. [Article in Portuguese] doi: 10.36660/abc.20210655 (PMC9363061; doi:10.36660/abc.20210655)
Supplement: Translation

# Translation: “Lightning The Heart” with 3D Echo: Transillumination of a Prosthetic Mitral Valve Dehiscence

**Authors:** Mariana Ribeiro Silva, Ana Isabel Azevedo, Francisco Sampaio, José Ribeiro, Ricardo Fontes-Carvalho

**Affiliations:** 1 Porto Portugal Centro Hospitalar de Vila Nova de Gaia/Espinho, Porto – Portugal; 2 Oporto Portugal University of Oporto,Oporto – Portugal

**Keywords:** Echocardiography, Three-Dimensional/méthods, Cardiovascular Diseases/diagnostic imaging, Cardiovascular Diseases/physiopathology, Image Interpretation, Computer Assisted, Observer, Variation, Transillumination, Mitral Valve/diagnosis

Three-dimensional (3D) echocardiogram with transillumination (TI) is a novel 3D rendering tool that enhances specific image features not optimally displayed by conventional 3D imaging. Conventional 3D rendering lacks consistency regarding image details and depth perception, often presenting suboptimal images. Transillumination 3D imaging benefits from the integration of a movable virtual light source into the data set. The light source can be moved anteriorly or posteriorly and from side to side, being positioned at specific locations in order to highlight the region of interest, increase accuracy, improve depth perception, create shadows, and enable a more precise distinction between structures. Additionally, this new 3D rendering tool improves visualization and delineation of orifice and edges, cavities, masses, and structural abnormalities,^1,2^ and is essential to perform detailed imaging during procedures.^3^

Transillumination may be particularly valuable in challenging scenarios, especially in the evaluation of cardiac prosthesis and devices that produce acoustic shadowing, leading to increased diagnostic accuracy.^4,5^

In the panel, we present two cases where 3D echocardiogram with TI conveyed additional diagnostic value in the evaluation of prosthetic valve dehiscence.

## Case 1

A 55-year-old man underwent mitral valve replacement with a caged-ball prosthesis (3M Starr-Edwards) at the age of 29. He was admitted with acute heart failure, functional New York Heart Association (NYHA) class III. Transthoracic echocardiography (TTE) showed a moderate-severe mitral periprosthetic leak; 3D-transesophageal echocardiography (TOE) showed prosthesis dehiscence, with a severe periprosthetic leak (Figure 1A). TI improved depth perception and accurate definition of the degree of prosthetic dehiscence, demonstrating a disinsertion of the mitral prosthesis, involving more than 50% of mitral circumference (Figure 1B; Video 1).

**Figure 1:** Panels A and B. 3M Starr-Edwards mitral prosthesis dehiscence viewed from left atrium (left atrial appendage at 9 o’clock position). Panel A: conventional 3D imaging. Panel B: TI rendering; the light is positioned below left atrial appendage. The shadowing effect improves depth perception and most accurate definition of the degree of prosthetic dehiscence. Panels C and D: Mitral ring dehiscence viewed from left atrium (aortic valve between 11 and 12 o’clock position). Panel C: conventional 3D imaging. Panel D: TI rendering; the light is positioned laterally near the left atrial appendage, enhancing the points of separation of the ring and depicting the integrity of the posterior mitral leaflet. 3D: three-dimensional; TI: transillumination.

**Video 1:** URL: http://abccardiol.org/supplementary-material/2022/11902/2021-0655-video-1.mp4

## Case 2

A 73-year-old man underwent mitral annuloplasty with a 34-mm complete ring. Four months later, he presented dyspnoea. TTE showed moderate mitral regurgitation (MR) and a hyperechogenic structure in the left atrium; 3D-TOE confirmed the presence of moderate MR and showed a partially detached ring (Figure 1C). In this case, TI enabled better visualization of the separation points of the prosthetic ring, showing the integrity of the posterior mitral leaflet, and provided additional information about MR mechanism, which was due to a dilated native mitral annulus that led to incomplete leaflet coaptation (Figure 1D; Video 2).

**Video 2:** URL: http://abccardiol.org/supplementary-material/2022/11902/2021-0655-video-2.mp4

Despite 3D TI being highly feasible in a variety of cardiac conditions, including structural heart disease, it is not a widely available technique yet, and it still requires adequate training and further studies focusing on clinically relevant endpoints and effectiveness to validate the implementation of TI rendering in routine clinical practice. Furthermore, the current evidence is still limited regarding the added benefit of TI when compared to other techniques.

Nevertheless, this novel technique does not require a steep learning curve and is a relatively intuitive process to move the virtual light source in order to emphasize the structure of interest.^4^ Transillumination rises as an alternative to conventional 3D imaging, especially in conditions where it is anticipated that conventional rendering will provide suboptimal images, particularly in the evaluation of prosthetic valve disease.^5^

These two cases highlight the importance of TI rendering in the evaluation of complex structural heart disease.
